# Induction of Expandable Adipose-Derived Mesenchymal Stem Cells from Aged Mesenchymal Stem Cells by a Synthetic Self-Replicating RNA

**DOI:** 10.3390/ijms19113489

**Published:** 2018-11-06

**Authors:** Chika Miyagi-Shiohira, Yoshiki Nakashima, Naoya Kobayashi, Shinji Kitamura, Issei Saitoh, Masami Watanabe, Hirofumi Noguchi

**Affiliations:** 1 Department of Regenerative Medicine, Graduate School of Medicine, University of the Ryukyus, 207 Uehara, Nishihara, Okinawa 903-0215, Japan; chika@med.u-ryukyu.ac.jp (C.M.-S.); nakasima@med.u-ryukyu.ac.jp (Y.N.); 2 Department of Surgery, Okayama Saidaiji Hospital, Okayama 704-8192, Japan; n-kobayashi@saidaiji-hp.or.jp; 3 Department of Nephrology, Rheumatology, Endocrinology and Metabolism, Okayama University Graduate School of Medicine, Dentistry and Pharmaceutical Sciences, Okayama 700-8558, Japan; kitamura@okayama-u.ac.jp; 4 Division of Pediatric Dentistry, Graduate School of Medical and Dental Science, Niigata University, Niigata 951-8514, Japan; isaito@dent.niigata-u.ac.jp; 5 Department of Urology, Okayama University Graduate School of Medicine, Dentistry and Pharmaceutical Sciences, Okayama 700-8558, Japan; masami5@md.okayama-u.ac.jp

**Keywords:** induced tissue-specific stem (iTS) cells, induced pluripotent stem (iPS) cells, reprogramming factors, adipose-derived mesenchymal stem cells (ADSCs), synthetic self-replicative RNA

## Abstract

Adipose-derived mesenchymal stem cells (ADSCs) have attracted attention due to their potential for use in the treatment of various diseases. However, the self-renewal capacity of ADSCs is restricted and their function diminishes during passage. We previously generated induced tissue-specific stem cells from mouse pancreatic cells using a single synthetic self-replicating Venezuelan Equine Encephalitis (VEE)-reprogramming factor (RF) RNA replicon (SR-RNA) expressing the reprogramming factors POU class 5 homeobox 1 (OCT4), Krueppel-like factor 4 (KLF4), Sex determining region Y-box 2 (SOX2), and Glis Family Zinc Finger 1 (GLIS1). This vector was used to generate induced pluripotent stem (iPS) cells. Here, we applied this SR-RNA vector to generate human iTS cells from aged mesenchymal stem cells (hiTS-M cells) deficient in self-renewal that were derived from adipose tissue. These hiTS-M cells transfected with the SR-RNA vector survived for 15 passages. The hiTS-M cells expressed cell surface markers similar to those of human adipose-derived mesenchymal stem cells (hADSCs) and differentiated into fat cells and osteoblasts. Global gene expression profiling showed that hiTS-M cells were transcriptionally similar to hADSCs. These data suggest that the generation of iTS cells has important implications for the clinical application of autologous stem cell transplantation.

## 1. Introduction

Bone marrow-derived mesenchymal stem cells and adipose-derived mesenchymal stem cells (ADSCs) have attracted attention because they have the potential for use in the treatment of various diseases. Numerous published clinical studies have described their use in lower limb revascularization, liver reproduction, cartilage reproduction, and treatment of graft-versus host disease [1,2,3,4,5,6,7,8,9,10,11,12,13,14,15]. However, the self-renewal competence of ADSCs is limited, and their function diminishes during passage [16,17,18]. In contrast, embryonic stem (ES) cells and induced pluripotent stem (iPS) cells self-renew and are pluripotent because they differentiate into cells derived from the three embryonic germ layers [19,20,21,22,23,24]. Numerous problems are associated with ES/iPS cell therapy. For example, ES cells may pose ethical issues, and ES and iPS cells may form tumors and have limited in vitro abilities to generate pure populations of differentiated cell types [25]. Therefore, we focused on developing a method for generating induced tissue-specific stem (iTS) cells derived from the pancreas (iTS-P) or liver (ITS-L) by transfecting them with a plasmid that coexpresses the reprogramming factors octamer-binding transcription factor 4 (OCT4), sex-determining region Y-box 2 (SOX2), kruppel-like factor 4 (KLF4), and c-MYC and then performing tissue-specific selection. Notably, the iTS-P/iTS-L cells were unable to generate teratomas when subcutaneously transplanted into immunodeficient mice.

iTS-P and iTS-L cells express genetic markers of pancreatic and hepatic progenitors, respectively. Such cells can be induced to differentiate into insulin-producing cells or hepatocytes, respectively, more frequently than ES cells [26,27]. We generated iTS-P cells using a single synthetic, self-replicating Venezuelan Equine Encephalitis (VEE)-reprogramming factor (RF) RNA replicon (SR-RNA) coexpressing the reprogramming factors OCT4, KLF4, SOX2, and Glis Family Zinc Finger 1 (GLIS1) [28,29].

Here, we applied this SR-RNA vector to generate expandable human iTS cells from aged mesenchymal stem cells (hiTS-M cells) derived from adipose tissue cells deficient in self-renewal.

## 2. Results

### 2.1. Generation of hiTS-M Cells from Human ADSCs by an RNA Expression Vector

**Figure 1:**
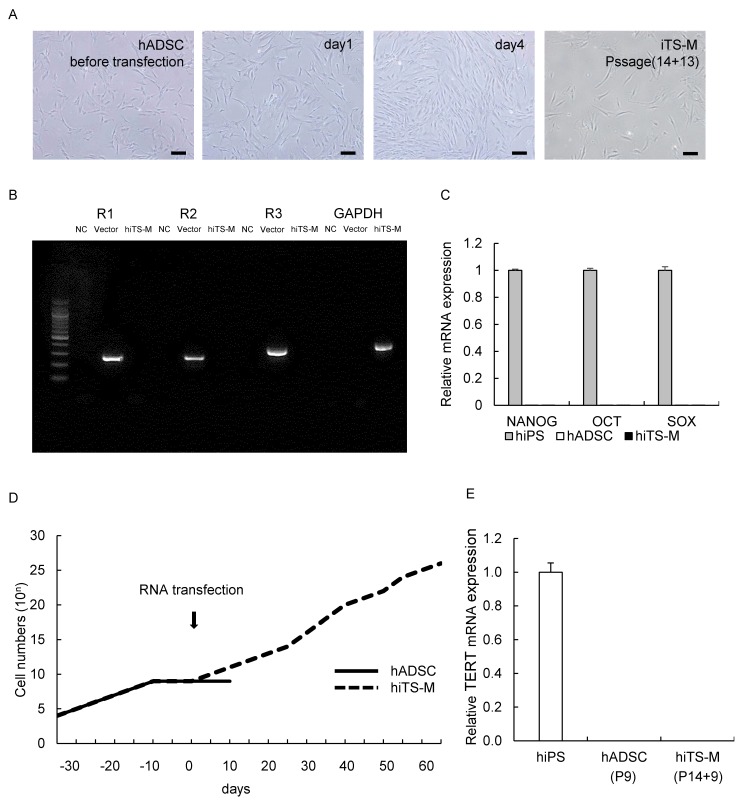
Generation of hiTS-M cells using a synthetic self-replicating RNA. (**A**) Morphology of hADSCs (passage 14) before transfection, one and four days after transfection, and hiTS-M (passage 14 + 13). Scale bars = 100 µm. (**B**) RT-PCR analysis of persistent VEE-RF RNA Replicon in hiTS-M cells. Total RNA from hiTS-M cells was prepared from passage (14 + 5). Reactions without template served as negative control (NC). VEE-RF RNA Replicon was used as a positive control (Vec). Locations of R1, R2, and R3 are shown in Figure S1A. Amplicon sizes: R1 = 302 bp, R2 = 302 bp, R3 = 394 bp, *GAPDH* = 452 bp. (**C**) qRT-PCR analysis of *NANOG*, *OCT4*, and *SOX2* expression, which are markers of ES/iPS cells, in hiPS cells (passage 20), hADSCs (passage 5), and hiTS-M cells (passage 14 + 5). Data are expressed as *NANOG-*, *OCT4-*, and *SOX2*-to-*GAPDH* ratios, with the ratio of iPS cells arbitrarily defined as one (*n* = 3). Error bars represent the standard error. (**D**) Growth curves of hADSCs (passage 9 to 14) and hiTS-M cells (passage 14 +and 0 to 15). (**E**) qRT-PCR analysis of *hTERT* expression in hiPS cells (passage 20), hADSCs (passage 9), and hiTS-M cells (passage 14 + 9). Data are expressed as *hTERT*-to-*GAPDH* ratios, with ratio of iPS cells arbitrarily defined as one (*n* = 3).

We attempted to generate hiTS-M cells from aged human ADSCs (hADSCs) that did not proliferate, cultured for 10 days after the 14th passage (Figure 1D). We transfected the aged hADSCs with the SR-RNA encoding the reprogramming factors OCT4, KLF4, SOX2, and GLIS1 (Figure S1). Aged hADSCs gradually proliferated until four days after transfection with the RNA vector (Figure 1A). Nine days after transfection, the proliferating hiTS-M cells were separately passaged in a new T25 flask. The hiTS-M cells exhibited a fibroblast-like morphology similar to that of hADSCs self-renewed for 15 additional passages, for a total of 29 passages (Figure 1A). In contrast, aged untransfected hADSCs did not proliferate (Figure 1D). Reverse transcription-polymerase chain reaction (RT-PCR) analysis did not detect the SR-RNA in hiTS-M cells at passage five after transfection (total passages: 14 + 5 = 19) (Figure 1B). We performed quantitative RT-PCR (qRT-PCR) analysis of marker genes of ES cells to investigate gene expression in hiTS-M cells (passage 29) and hADSCs (passage five). We were unable to detect the mRNAs encoding the pluripotency markers NANOG, OCT4, or SOX2 in either cell type (Figure 1C). We evaluated telomerase activity in hiTS-M cells. Both hADSCs (passage 9) and hiTS-M cells (passage 14 + 9) did not express human telomerase reverse transcriptase (*hTERT*) (Figure 1E).

### 2.2. Characterization of hiTS-M Cells Transfected with the RNA Vector

**Figure 2:**
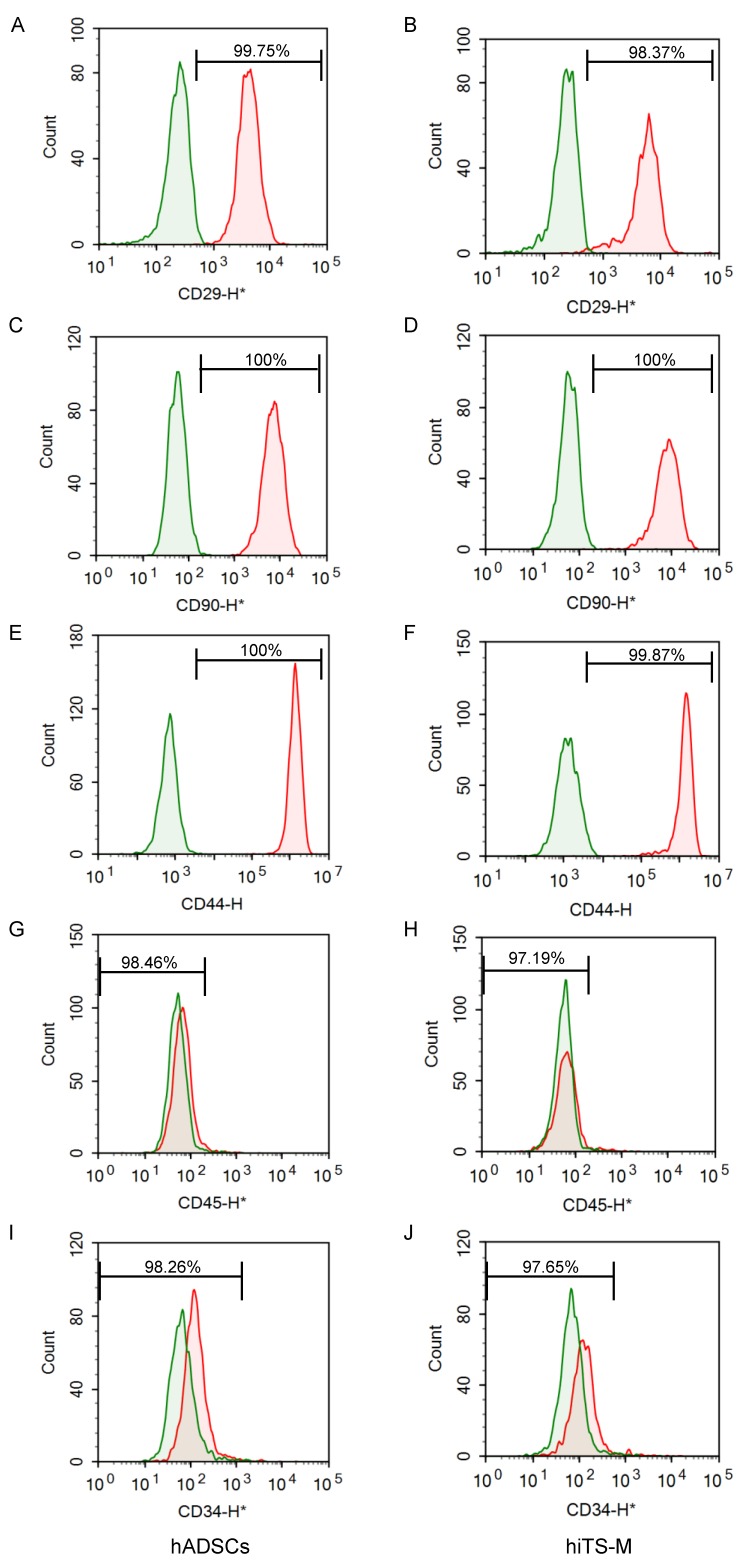
Flow cytometric analysis. hiTS-M cells (passage 14 + 7) and hADSCs (passage 7) were analyzed: (**A**) hADSCs, CD29; (**B**) hiTS-M cells, CD29; (**C**) hADSCs, CD90; (**D**) hiTS-M cells, CD90; (**E**) hADSCs, CD44; (**F**) hiTS-M cells, CD44; (**G**) hADSCs, CD45; (**H**) hiTS-M cells, CD45; (**I**) hADSCs, CD34; and (**J**) hiTS-M cells, CD34.

We performed flow cytometry to detect cell surface markers characteristic of hADSCs that were expressed by hiTS-M cells. The hiTS-M cells (passage 14 + 7) and hADSCs (passage 7) expressed integrin β-1 (CD29) at 99.75% and 98.37%, respectively; Thy-1 (CD90) (each 100%); and hyaluronate receptor/phagocytic glycoprotein-1 (CD44) at 100 and 99.87%, respectively (Figure 2A–F). The hiTS-M cells and hADSCs rarely expressed protein tyrosine phosphatase, receptor type (CD45) (1.54% and 2.81%, respectively) and leukocyte common antigen (CD34) (1.74% and 2.35%, respectively) (Figure 2G–J). These data suggest that hiTS-M cells expressed hADSC surface markers.

### 2.3. Genes and Proteins Expressed in hiTS-M Cells

**Figure 3:**
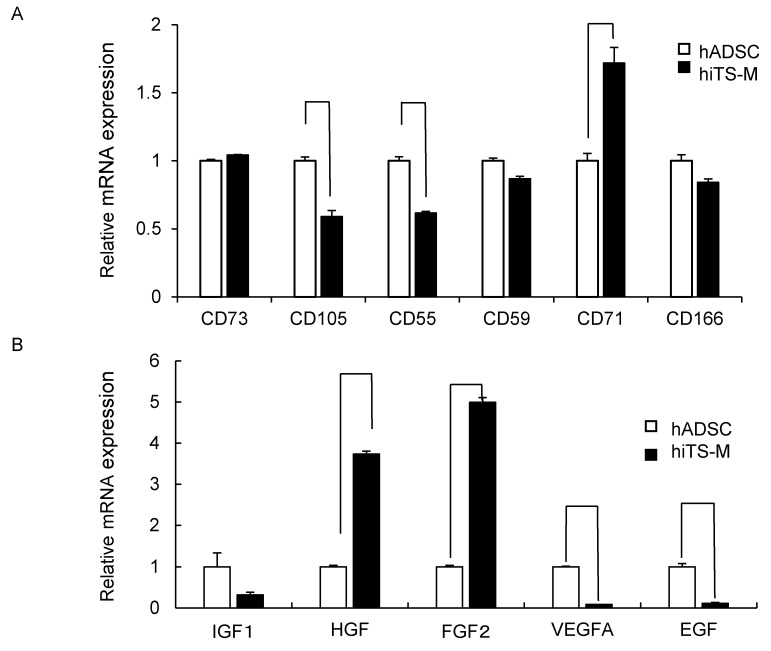

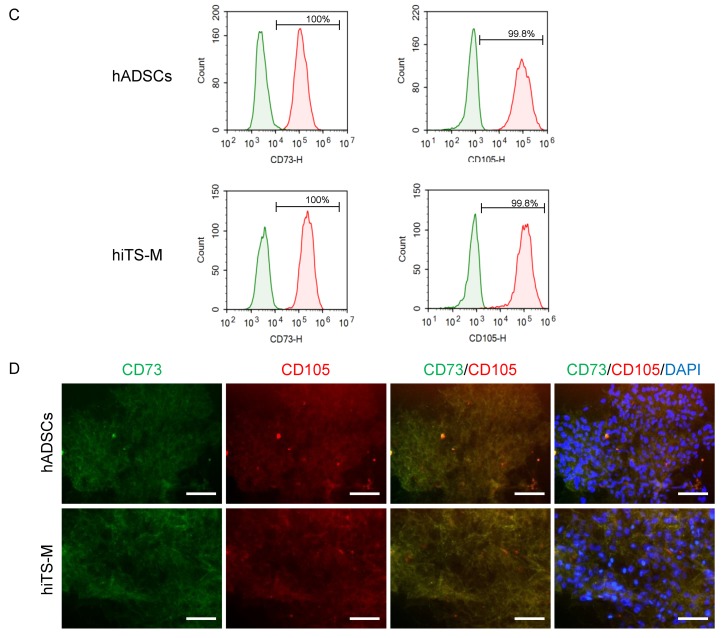
Genes and proteins expressed in hiTS-M cells. (**A**) qRT-PCR analysis of expression of genes encoding cell surface markers of hiTS-M cells. hADSCs were used as a control. (**B**) qRT-PCR analysis of expression of marker genes encoding growth factors produced by hiTS-M cells. hADSCs were used as a control. hiTS-M cells (passage 14 + 7) and hADSCs (passage 7) were used. Data are expressed as mRNA-to-*GAPDH* mRNA ratio, with the ratio of control cells arbitrarily defined as at one (*n* = 3). Error bars represent the standard error. * *p* < 0.01. (**C**) Flow cytometric analysis of CD73 and CD105. hiTS-M cells (passage 14 + 7) and hADSCs (passage 7) were analyzed. (**D**) Immunofluorescence of CD73 and CD105 in hADSCs and hiTS-M cells. Scale bars = 100 µm.

We investigated the mRNAs encoding CD73, CD105, CD55, CD59, CD71, and CD166, which are specific markers for ADSCs. hiTS-M cells (passage 14 + 6) and hADSCs (passage 6) expressed each mRNA, and the hiTS-M cells expressed significantly higher levels of *CD71* mRNA. In contrast, hiTS-M cells expressed significantly lower levels of *CD105* and *CD55* mRNAs than hADSCs (Figure 3A). hiTS-M cells and hADSCs expressed the mRNAs encoding insulin-like growth factor 1 (IGF1), hepatocyte growth factor (HGF), fibroblast growth factor 2 (FGF2), vascular endothelial cell growth factor A (VEGFA), and epidermal growth factor (EGF). hiTS-M cells expressed *HGF* and *FGF2* at levels four- and six-fold higher compared with hADSCs, respectively. In contrast, hiTS-M cells expressed significantly lower levels of *VEGFA* and *EGF* mRNAs compared with hADSCs (Figure 3B).

We also investigated expression of CD73 and CD105 protein by Flow cytometry and immunofluorescence. Both hADSCs and hiTS-M cells expressed CD73 and CD105 protein (Figure 3C,D).

Kumar et al. showed that mesenchymal progenitors derived from human pluripotent stem cells give rise to proliferative pericytes, smooth muscle cells, and mesenchymal stem/stromal cells [9]. We evaluated which cell types hiTS-M cells included. Over 99% of hiTS-M cells did not express NG2, Calponin, or Desmin, similar to hADSCs (Figure S2). Therefore, over 99% of hiTS-M cells were mesenchymal stem/stromal cells.

### 2.4. Analysis of the Differentiation Potential of hiTS-M Cells

**Figure 4:**
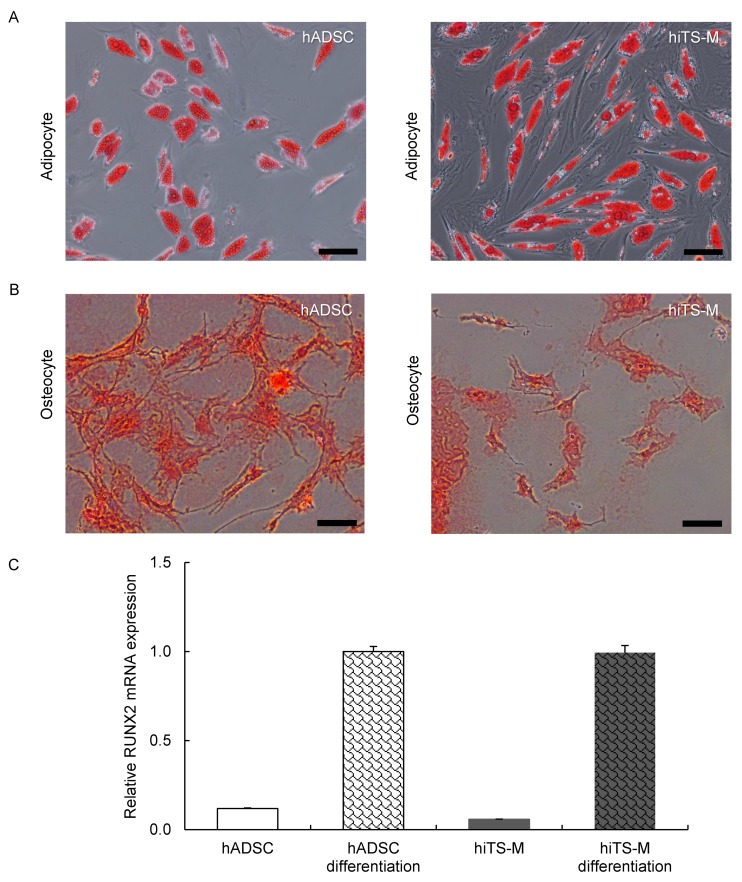
Differentiation of hiTS-M cells into adipocytes and osteocytes. Differentiation of hADSCs and hiTS-M cells into (**A**) adipocyte and (**B**) osteocytes. hADSCs (passage 3) and hiTS-M cells (passage 14 + 4) were used. Scale bars = 100 µm. (**C**) qRT-PCR analysis of *RUNX2* expression in hADSCs (passage 5) and hiTS-M cells (passage 14 + 5). Data are expressed as *RUNX2*-to-*GAPDH* ratios, with the ratio of differentiated hADSCs arbitrarily defined as one (*n* = 3).

To test whether the hiTS-M cells underwent adipogenic differentiation, the cells were treated with adipogenic induction medium for seven days and cultured in maintenance medium for an additional seven days. Oil Red O stained all hiTS-M cells (Figure 4A), suggesting that they exhibited the adipogenic phenotype.

To determine whether the hiTS-M cells underwent osteogenic differentiation, the cells were cultured in osteogenic induction medium for 10 days. All hiTS-M cells possessed calcified nodules revealed by staining with Alizarin Red solution (Figure 4B), suggesting that the treated hiTS-M cells exhibited the osteogenic phenotype.

### 2.5. Bisulfite Sequencing of the Genomic Promoter Regions of OCT4 and NANOG in hiPS Cells, hADSCs, and hiTS-M Cells

**Figure 5:**
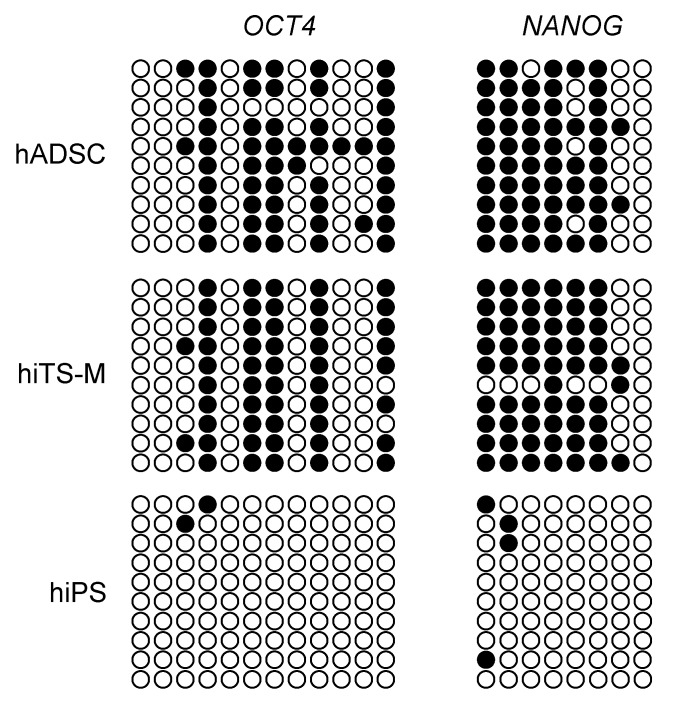
Bisulfite genomic sequencing of promoter regions of *OCT4* and *NANOG* in hADSCs, hiTS-M cells, and hiPS cells. Open circles indicate unmethylated CpG dinucleotides, whereas closed circles indicate methylated CpGs.

Bisulfite genomic sequencing demonstrated that the promoters of *OCT4* and *NANOG* were methylated in hiTS-M cells (passage 14 + 6) and hADSCs (passage 6). In contrast, these promoters were almost completely demethylated in hiPS cells (passage 20) (Figure 5). These results demonstrate that the extents of methylation of these promoters in hiTS-M cells were similar to those of hADSCs and differed from those of hiPS cells.

### 2.6. Microarray Analysis

**Figure 6:**
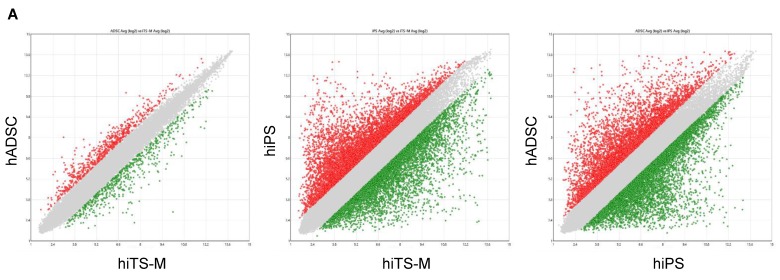

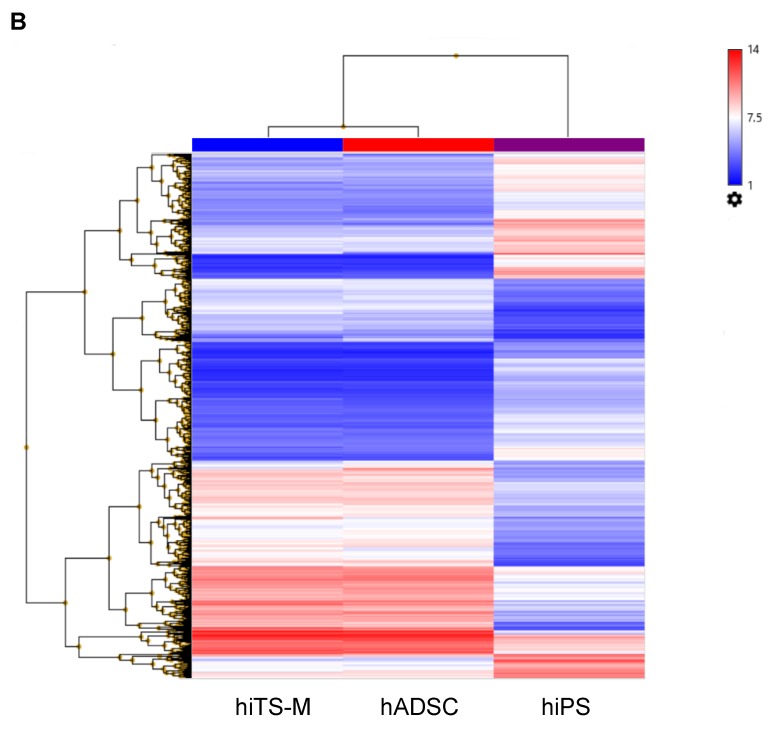
Microarray analysis. (**A**) A Transcriptome Analysis Console was used to analyze global gene expression patterns between hiPS cells and hiTS-M cells, between hADSCs and hiTS-M cells, and between iPS cells and hADSCs. Grey area indicates genes expressed at levels less than two-fold different between the two samples. (**B**) Unsupervised hierarchical clustering of gene expression profiles of hiPS cells, hiTS-M cells, and hADSCs. Each column represents one biological sample.

We compared the global gene expression profiles of hiPS cells (passage 20), hADSCs (passage 6), and hiTS-M cells (passage 14 + 6) (Table S3). Of 54,613 genes, 2.13% differed by more than two-fold between hADSCs and hiTS-M cells; 19.53% differed by more than two-fold between hiPS cells and hiTS-M cells; and 20.78% differed by more than two-fold between hiPS cells and hADSCs (Figure 6A). These data suggest that the expression pattern of hiTS-M cells was similar to that of hADSCs but differed from that of hiPS cells. Unsupervised hierarchical clustering of the gene expression profiles of hiTS-M cells, hADSCs, and hiPS cells showed that hiTS-M cells clustered more closely with hADSCs than hiPS cells (Figure 6B).

## 3. Discussion

Here, we generated hiTS-M cells using an SR-RNA vector capable of expressing the reprogramming factors OCT4, KLF4, SOX2, and GLIS1. The RNA vector was originally utilized to generate iPS cells [28]. Because hADSC culture medium was used after transfection, iPS cells were not generated in this study. Epigenetic memory is inherited from parental cells following the reprogramming of iPS cells, and the phenotypes of iPS cells may be influenced by the parental cells [30,31,32,33,34,35]. Although some of the aged hADSCs may have induced hiPS cells immediately after transfection in this study, the cells could not maintain the hiPS cell phenotype because the culture conditions were suitable for propagating hADSCs but not ES/iPS cells. Our present study shows that hiTS-M cells were generated by transient overexpression of reprogramming factors combined with hADSC culture conditions.

ADSCs have a limited self-renewal capacity, and their maximum number of passages is normally 10–15 [36,37]. We showed here that the hADSCs could be passaged 15 times after transfection (14 passages before transfection and 15 passages after transfection), and the cell number increased from 10^9^ (day 0) to 10^26^ (day 70) (Figure 1D). Extending the passage number is important for autologous transplantation of ADSCs. In contrast, the characteristics of hiTS-M cells were similar but not identical to those of hADSCs as indicated by qRT-PCR and microarray analyses. The levels of mRNAs encoding CD105, CD55, VEGFA, and EGF were lower in hiTS-M cells compared with those of hADSCs, whereas those of the mRNAs encoding CD71, HGF, and FGF2 were higher in hiTS-M cells compared with those of hADSCs. This may be explained by the degree of reprogramming induced by the proteins expressed by the SR-RNA vector.

In this study, we generated human iTS-M cells using a single, synthetic, self-replicating VEE-RF RNA replicon. Other groups, including ours, have used plasmid transfection to generate iPS/iTS cells [26,27]. However, these showed a DNA integration of the plasmid of around 10–20%. RNA vector was used in this study to avoid potential integration problems.

The characteristics of ADSCs change during passage [36,37]. When aged hADSCs are strongly reprogrammed by the high expression of reprogramming factors, the characteristics of the hiTS-M cells may be similar to those of hADSCs during early passages. When aged hADSCs are weakly reprogrammed by the low expression of reprogramming factors, the characteristics of the hiTS-M cells may be similar to those of late passage hADSCs. The characteristics of hiTS-M cells may depend on the degree of reprogramming and their passage number after transfection. As shown in Figure 4, both hiTS-M and hADSCs appear to have partially lost the ability to differentiate. This may be due to the variability in hADSCs and in the reprogramming of hiTS-M cells.

In conclusion, we generated hiTS-M cells from aged hADSCs. hiTS-M cells expressed cell surface markers similar to those of hADSCs and were induced to differentiate into fat cells and osteoblasts. qRT-PCR and bisulfite genomic sequencing analyses of *OCT4* and *NANOG* showed that the phenotype of hiTS-M cells differed significantly from that of hiPS cells. Global gene-expression profiling showed that hiTS-M cells are transcriptionally similar to hADSCs. Because the self-renewal capacity of hADSCs is restricted and the function of hADSCs decreases during passage, the generation of hiTS-M cells may have important implications for the clinical application of autologous stem cell transplantation.

## 4. Materials and Methods

### 4.1. Culture of hADSCs

Human ADSCs derived from a 40-year-old female donor (Lot No. 0000353102; body mass index, 23; passage 1) were obtained from Lonza Japan, Inc. (Tokyo, Japan). For adherent cell culture, 4 × 10^5^ hADSCs were seeded into a T-25 flask (Asone, Osaka, Japan) using the Poietics ADSC-GM BulletKit (PT-4505; Lonza Japan) at 37 °C in a humidified atmosphere containing 5% CO_2_. The cells were cultured until they were approximately 80–90% confluent. The cells were detached from the culture flask using trypsin-ethylenediaminetetraacetic acid (EDTA) (0.25% *w*/*v*; Wako, Osaka, Japan) and repeatedly seeded and cultured in new culture flasks. The hADSCs were passaged 14 times until they failed to proliferate. The hADSCs, from passages four to seven, were used as a control for the analyses as follows: RT-PCR, qRT-PCR, differentiation into adipocytes and osteocytes, bisulfite genomic sequencing, and microarray analysis.

### 4.2. Generation of hiTS-M Cells Using an RNA Replicon

The hiTS-M cells were generated as described previously [28], with minor modification, using a Simplicom RNA Reprogramming Kit (Merck Millipore, Tokyo, Japan). Aged hADSCs (passage 14) were seeded into a T25 plate on day-10 (40–50% confluency). To minimize the interferon response, cells were treated with 1 mL of Advanced DMEM (Thermo Fisher Scientific, Waltham, MA, USA) containing 0.2 µg of B18R protein (Merck Millipore, Burlington, MA, USA) 2 h before transfection. On day 0, 0.5 µg VEE-OKS-iG and 0.5 µg B18R mRNAs was used to transfect cells in the presence of Lipofectamine 2000 (Thermo Fisher Scientific). After 3 h, the transfection medium was changed to Advanced DMEM containing 200 ng/mL of B18R protein. The cells were passaged on day 9, and Advanced DMEM containing 200 ng/mL of B18R protein was supplied every day until day 16. Cells were then passaged on day 16 and Advanced DMEM was replaced with hADSC culture medium (Figure S1).

### 4.3. RT-PCR and qRT-PCR Analyses

Total RNA was extracted from cells using an RNeasy Mini Kit (Qiagen, Tokyo, Japan). After quantifying the RNA using spectrophotometry, 2.5 µg RNA was heated at 85 °C for 3 min and then reverse-transcribed in a 25-µL solution containing 200 units of Superscript II RNase H-RT (Thermo Fisher Scientific), 50 ng random hexamers (Thermo Fisher Scientific), 160 µmol/L dNTP, and 10 nmol/L dithiothreitol. The reaction conditions were as follows: 10 min at 25 °C, 60 min at 42 °C, and 10 min at 95 °C. Amplifications were performed using a Perkin-Elmer 9700 Thermocycler (Perkin-Elmer, Waltham, CT, USA) with 3 µL of cDNA (20 ng DNA equivalents), 160 µmol/L of cold dNTPs, 10 pmol of appropriate oligonucleotide primers, 1.5 mmol/L of MgCl_2_, and 5 units of AmpliTaq Gold DNA polymerase (Perkin-Elmer, Norwalk, CT, USA) in 1× PCR buffer. The thermal cycle profile was as follows: 10 min denaturation at 94 °C followed by an amplification cycle (1 min denaturation at 94 °C, 1 min annealing at 57 °C to 62 °C, and a 1 min extension at 72 °C) with a final extension step of 10 min at 72 °C. The oligonucleotide primers are shown in Table S1.

The mRNAs were quantified using a TaqMan Real-time PCR System (Applied Biosystems, Foster City, CA, USA) according to the manufacturer’s instructions. PCR was performed for 40 cycles, including 2 min at 50 °C and 10 min at 95 °C as initial steps. In each cycle, denaturation was performed for 15 s at 95 °C, and annealing and extension were performed for 1 min each at 60 °C. PCR was performed in 20 µL of a solution of cDNAs synthesized from 1.11 ng of total RNA. The level of each mRNA sample was normalized by dividing its amount by that of *GAPDH* mRNA. Primers for the mRNAs encoding OCT4, SOX2, NANOG, TERT, 5′-nucleotidase (CD73), endoglin (CD105), complement decay accelerating factor (CD55), complement protectin (CD59), transferrin receptor (CD71), and activated lymphocyte cell adhesion molecule (CD166); primers specific for the genes encoding IGF1, HGF, FGF2, VEGFA, EGF, RUNX2, and GAPDH were included in Assays-on-Demand Gene Expression Products (Thermo Fisher Scientific).

### 4.4. Flow Cytometry

Flow cytometry was performed using a NovoCyte Flow Cytometer (ACEA Biosciences, Inc., San Diego, CA, USA) according to the manufacturer’s instructions. Briefly, hADSCs (1 × 10^5^ cells) were suspended in 0.5 mL phosphate-buffered saline (PBS) (Wako, Osaka, Japan). Each antibody (1/100 volume) was added to the cell suspensions and incubated on ice for 30 min. Analyses were performed after washing the cells with Brilliant Stain Buffer (BD Biosciences, Franklin Lakes, NJ, USA). Primary antibodies were as follows: Allophycocyanin (APC) Mouse Anti-Human, CD29, BV421 Mouse Anti-Human CD44, BV421 Mouse IgG2b κ Isotype Control, APC Mouse IgG1 κ Isotype Control (BD Biosciences); FITC anti-human CD90.2 (Thy1) Antibody, FITC Mouse IgG1 κ Isotype Ctrl Antibody, PerCP anti-human CD34 Antibody, PerCP Mouse IgG1 κ Isotype Ctrl Antibody, PE/Cy7 anti-human CD45 Antibody, PE/Cy7 Mouse IgG1 κ Isotype Ctrl Antibody, FITC anti-human CD73 (Ecto-5′-nucleotidase) Antibody, and Alexa Fluor 647 anti-human CD105 Antibody (BioLegend, Inc., San Diego, CA, USA) [38].

### 4.5. Immunostaining

Cell were fixed with 4% paraformaldehyde in PBS buffer. After blocking with 20% AquaBlock (EastCoast Bio, Inc. North Berwick, ME, USA) for 30 min at room temperature, the cells were incubated overnight at 4 °C with each 1st antibody (FITC anti-human CD73 (Ecto-5′-nucleotidase) Antibody, Alexa Fluor 647 anti-human CD105 Antibody, Alexa Fluor 488 anti-human NG2 Antibody, mouse anti-human Calponin Antibody, and rabbit anti-Desmin Antibody) and then for 1 h at room temperature with each second antibody (AlexaFluor 488 anti-mouse antibody, AlexaFluor 488 anti-rabbit antibody). Mounting medium for fluorescence with DAPI (Vector Laboratories Inc., Burlingame, CA, USA) was used for mounting.

### 4.6. Cell Differentiation

Adipogenic differentiation was performed using Adipogenic Differentiation Medium (DM-2; Zen-Bio, Inc., Research Triangle Park, NC, USA) and Adipocyte Maintenance Medium (AM-1; Zen-Bio, Inc.). Confluent hiTS-M cells (passage 14 + 7) and hADSCs (passage 7) (three to four days after seeding into six-well plates using the designated medium) were cultured for seven days using Adipocyte Differentiation Medium (DM-2; Zen-Bio, Inc.) to induce differentiation into adipocytes. The medium was subsequently switched to Adipocyte Maintenance Medium (AM-1; Zen-Bio, Inc.) and changed every three days. Adipocytes with lipid droplets were detected using light microscopy after 20–30 days. Differentiation was confirmed by Oil Red O (Cosmo Bio Co., Ltd., Tokyo, Japan) staining of intracellular lipid droplets. Differentiated ASCs were fixed in 10% formaldehyde (Wako, Osaka, Japan) in PBS (Wako, Osaka, Japan) for at least 10 min, washed with 60% isopropanol (Wako, Osaka, Japan), and stained with Oil Red O (Wako, Osaka, Japan) for 10 min followed by repeated washing with water and destaining in 100% isopropanol for 1 min [14,25,39].

Osteogenic differentiation was induced by culturing the hiTS-M cells and hADSCs (passage 3) for 10 days in MSC Go Rapid Osteogenic XF^TM^ (Cosmo Bio Co., Ltd., Tokyo, Japan). Differentiation was determined through detection of extracellular matrix calcification as revealed by staining with Alizarin Red solution (ARD-A1; PG Research Co., Ltd., Tokyo, Japan) according to the manufacturer’s instructions [10,14,25,39,40].

### 4.7. Bisulfite Genomic Sequencing

Bisulfite treatment was performed using the CpGenome Turbo Bisulfite Modification Kit (Merck Millipore) according to the manufacturer’s recommendations. The PCR primers are listed in Table S2. Amplicons were cloned using a Mighty TA-cloning kit (TAKARA BIO INC., Shiga, Japan). Ten randomly-selected clones were sequenced using M13 forward and reverse primers specific for each gene.

### 4.8. Microarray Analysis

Total RNA from ES cells, iTS-P cells, or islets was labeled with biotin. Samples were hybridized to the GeneChip 3′IVT PLUS Reagent Kit (Affymetrix, Tokyo, Japan) and GeneChip Hybridization, Wash and Stain Kit (Affymetrix, Tokyo, Japan) according to the manufacturer’s protocol. Arrays were scanned using a GeneChip Scanner 3000 7G (Affymetrix, Tokyo, Japan). Data were analyzed using Transcriptome Analysis Console 4.0 software (Thermo Fisher).

### 4.9. Statistical Analyses

The data are expressed as the mean ± standard error. Two groups were compared using the Student *t*-test. The differences between each group were considered significant if *p* < 0.01. All statistical analysis methods were performed in accordance with the relevant guidelines and regulations.

## Supplementary Materials

The following are available online at http://www.mdpi.com/1422-0067/19/11/3489/s1.
