# Supplementary material for: Induction of Expandable Adipose-Derived Mesenchymal Stem Cells from Aged Mesenchymal Stem Cells by a Synthetic Self-Replicating RNA
Source: Int J Mol Sci. 2018 Nov 6;19(11):3489. doi: 10.3390/ijms19113489 (PMC6274871; doi:10.3390/ijms19113489)
Supplement: Supplementary file 1 [file ijms-19-03489-s001.zip › supplementary materials/Supplementary Materials.docx]

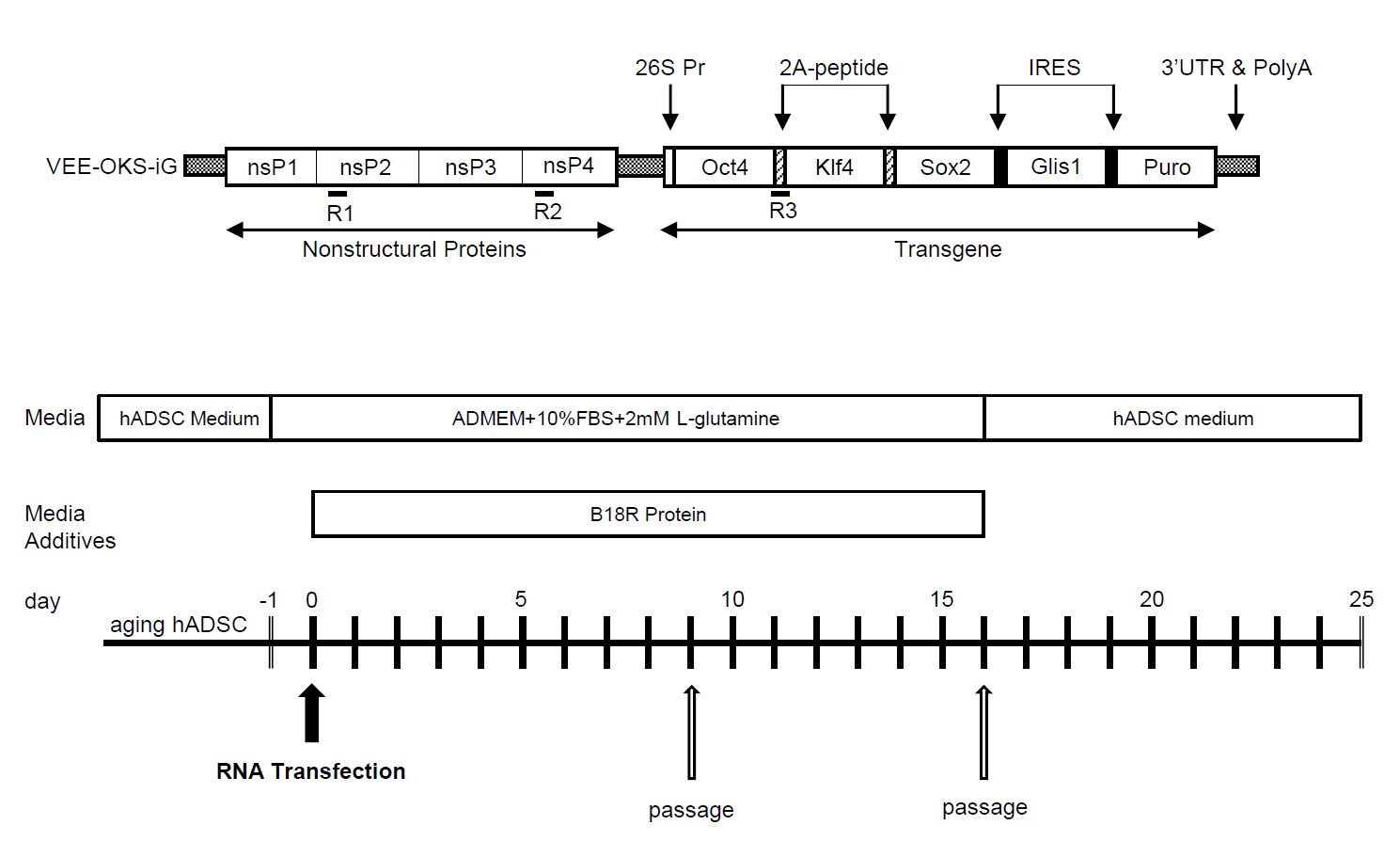


**Supplemental Figure S1. Vector and time schedules for the generation of human iTS-M cells from human ADSCs.** (A) Schematic illustration of the VEE-RF RNA replicons. 5’ end nsP1–nsP4, nonstructural proteins 1–4; 3’ end reprogramming factors (Oct3/4, Klf4, Sox2, and Glis1). Location of the 26S internal promoter, ribosome-shifting 2A peptide, IRES sequence, puromycin (Puro)-resistance gene, and PCR detection of replicon as indicated. (B) Time schedules for the induction of hiTS-M cells. Aged hADSCs (passage 14, 1 × 10^4^ cells) were plated on day –10 and cotransfected (Tfx) with the VEE-RF RNA replicon plus B18R mRNA on day 0. Cells were cultured in B18R-CM until day 16.


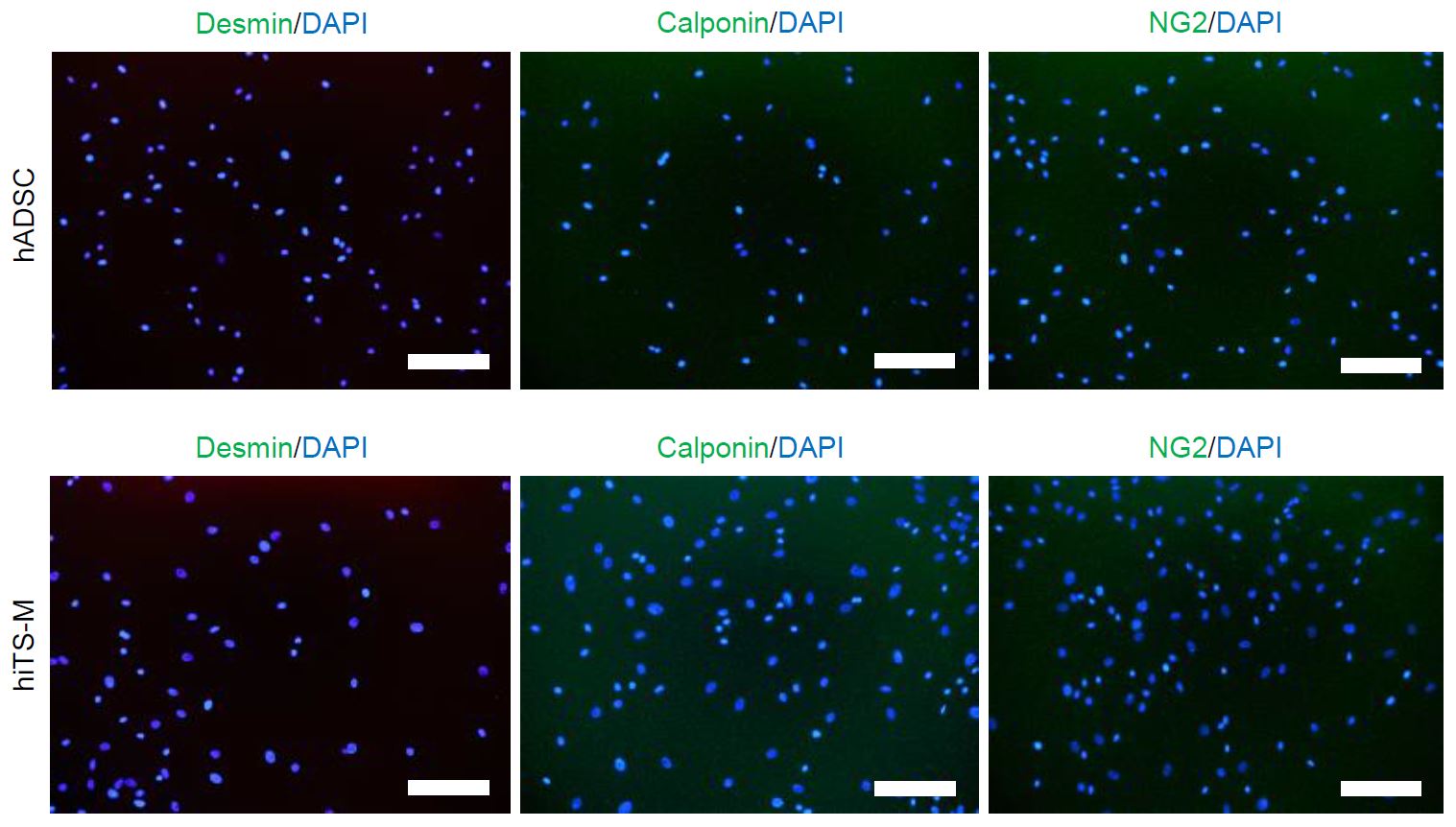
Supplemental Figure 2. Immunohistochemistry analysis of markers of smooth muscles cells and pericytes in hiTS-M cells. Nucrei (blue) were co-stained with DAPI. Scale bars = 100 µm

**Supplemental Table S1. Primers**

Forward Reverse

R1 caggacgatctcattctcac gcttgccactcctctatcgtg

R2 ccacaatacgatcggcagtg atgtcctgcaacatattcaaa

R3 cggcgccagaagggcaagcg cacctgcttgacgcagtgtc

GAPDH accacagtccatgccatcac tccaccaccctgttgctgta

**Supplemental Table S2. Primers**

Forward Reverse

OCT4 p gaggttggagtagaaggattgttttggttt cccccctaacccatcacctccaccacctaa

NANOG p tggttaggttggttttaaatttttg aacccacccttataaattctcaatta
